# Predicting protein functions using positive-unlabeled ranking with ontology-based priors

**DOI:** 10.1093/bioinformatics/btae237

**Published:** 2024-06-28

**Authors:** Fernando Zhapa-Camacho, Zhenwei Tang, Maxat Kulmanov, Robert Hoehndorf

**Affiliations:** Computational Bioscience Research Center (CBRC), King Abdullah University of Science and Technology, Thuwal, 23955-6900, Saudi Arabia; Computer, Electrical and Mathematical Sciences & Engineering Division (CEMSE), King Abdullah University of Science and Technology, Thuwal, 23955-6900, Saudi Arabia; Department of Computer Science, University of Toronto, Toronto, ON M5S 1A1, Canada; Computational Bioscience Research Center (CBRC), King Abdullah University of Science and Technology, Thuwal, 23955-6900, Saudi Arabia; Computer, Electrical and Mathematical Sciences & Engineering Division (CEMSE), King Abdullah University of Science and Technology, Thuwal, 23955-6900, Saudi Arabia; SDAIA-KAUST Center of Excellence in Data Science and Artificial Intelligence, King Abdullah University of Science and Technology, Thuwal, 23955-6900, Saudi Arabia; Computational Bioscience Research Center (CBRC), King Abdullah University of Science and Technology, Thuwal, 23955-6900, Saudi Arabia; Computer, Electrical and Mathematical Sciences & Engineering Division (CEMSE), King Abdullah University of Science and Technology, Thuwal, 23955-6900, Saudi Arabia; SDAIA-KAUST Center of Excellence in Data Science and Artificial Intelligence, King Abdullah University of Science and Technology, Thuwal, 23955-6900, Saudi Arabia

## Abstract

Automated protein function prediction is a crucial and widely studied problem in bioinformatics. Computationally, protein function is a multilabel classification problem where only positive samples are defined and there is a large number of unlabeled annotations. Most existing methods rely on the assumption that the unlabeled set of protein function annotations are negatives, inducing the *false negative* issue, where potential positive samples are trained as negatives. We introduce a novel approach named PU-GO, wherein we address function prediction as a positive-unlabeled ranking problem. We apply empirical risk minimization, i.e. we minimize the classification risk of a classifier where class priors are obtained from the Gene Ontology hierarchical structure. We show that our approach is more robust than other state-of-the-art methods on similarity-based and time-based benchmark datasets.

**Availability and implementation:**

Data and code are available at https://github.com/bio-ontology-research-group/PU-GO.

## 1 Introduction

Deciphering the functions of proteins is essential for unraveling the complexities of cellular pathways (Eisenberg *et al.* 2000), identifying potential drug targets (Schenone *et al.* 2013), and understanding diseases (Liu *et al.* 2015). In bioinformatics, protein function prediction emerges as a formidable challenge. With the rapid growth of biological data, including genomic and proteomic information, there is a pressing need for effective computational methods to predict protein functions accurately. Currently, the Uniprot Knowledge Base (UniprotKB) (Consortium 2022) contains more than 250 million protein sequences and only few of them have experimental functional annotations. The Gene Ontology (GO) (Ashburner *et al.* 2000) provides structured information about protein functions and describes more than 50 000 functions in three subontologies: Molecular Function Ontology (MFO), Cellular Component Ontology (CCO), and Biological Process Ontology (BPO).

Despite substantial progress in bioinformatics, the functional annotations of proteins remain incomplete. A significant portion of the proteome lacks detailed functional characterization, hindering our comprehensive understanding of cellular processes. This incompleteness stems from the limitations of experimental techniques and the resource-intensive nature of functional assays. As a result, computational methods play a pivotal role in filling these knowledge gaps and providing predictions for unannotated or poorly characterized proteins.

In the pursuit of accurate protein function prediction, many existing methods adopt a binary classification learning framework, optimizing classifiers using unlabeled protein-function annotations as negative samples. This traditional approach, while effective in certain contexts, overlooks the nuances inherent in the protein function prediction landscape. Unlabeled samples might hide positive protein function annotations yet to be discovered.

UniprotKB regularly introduces new annotations for proteins; for example, from UniprotKB version 2023_03 to UniprotKB version 2024_01, there were 2236 proteins that gained 4236 functional annotations. Protein functional annotations can be propagated using the true-path rule (Ashburner *et al.* 2000), which results in 31 149 propagated annotations that are added to UniprotKB from version 2023_03 to version 2024_01. The oversimplified binary approach may ignore the uncertainty of unlabeled annotations and erroneously guide the classifiers to predict false negatives.

Positive unlabeled (PU) learning represents a paradigm shift in addressing these challenges. PU learning acknowledges the inherent uncertainty in the functional status of unlabeled protein function annotations and recognizes them as potential positives. In the PU learning realm, there are various strategies handle unlabeled data (Bekker and Davis 2020) such as negative extraction from the unlabeled set (Fung *et al.* 2006), empirical risk minimization (du Plessis *et al.* 2014) or multiclassifier aggregation (Mordelet and Vert 2014).

PU learning has been applied to different bioinformatics tasks (Li *et al.* 2021) such as disease gene predictions (Yang *et al.* 2012, Vasighizaker and Jalili 2018, Stolfi *et al.* 2023), drug–target interaction prediction (Lan *et al.* 2016, Peng *et al.* 2017) as well as protein function prediction (Youngs *et al.* 2013, Song *et al.* 2021). There are two main strategies in which PU learning has been applied: negative extraction from the unlabeled data and probabilistic adaptation of a classifier (Li *et al.* 2021). Negative-extraction methods are a two-step process where a subset of *reliable negatives* are extracted from the unlabeled set and then a classifier is optimized with a conventional learning algorithm. Although this approach can show effectiveness across different bioinformatics tasks, the strategy of pre-selecting negatives can exclude important samples, producing inaccurate or biased classifiers.

Methods that adapt a classifier do not need to estimate a negative sample set *a priori*. Instead, the classifier is optimized with the whole dataset (positive and unlabeled) and estimation of positives/negatives from the unlabeled set are performed afterwards. These methods rely on the probabilistic formulation defined by (Elkan and Noto 2008) for PU learning.

In the context of function prediction, most methods follow the negative samples extraction strategy (Zhao *et al.* 2008, Chen *et al.* 2010, Youngs *et al.* 2013), meaning that training is done with a fraction of the given data. Conversely, methods that learn a classifier with PU data directly (Song *et al.* 2021) rely on optimization frameworks such as Majorization Minimization (Lange *et al.* 2000) or Support Vector Machines (Cortes and Vapnik 1995). However, in recent years, protein function prediction has been extensively addressed with emerging deep learning techniques(Kulmanov *et al.* 2017, Cao and Shen 2021, Wang *et al.* 2023, Yuan *et al.* 2023).

**Figure 1.**
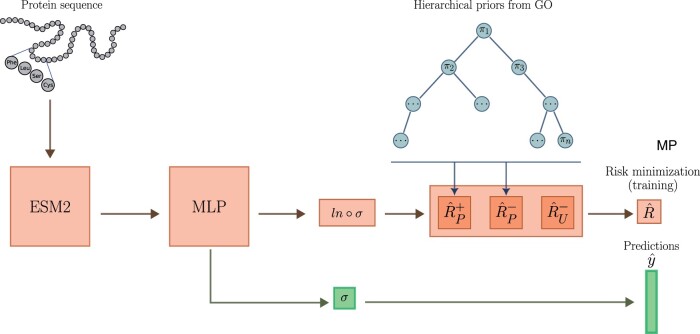
PU-GO workflow. The MLP classifier is trained to minimize classification risk of positive and unlabeled samples. Prior factors for each GO class is computed based on hierarchical GO structure.

We present PU-GO a method for predicting protein functions by optimizing a classifier under PU learning framework. Instead of pre-selecting negatives samples, PU-GO uses the classifier adaptation approach and minimizes classifications risks of positive and unlabeled samples (du Plessis *et al.* 2014). Our framework uses the ESM2 15B protein language model (Lin *et al.* 2023) to obtain high-dimensional feature vectors for protein sequences, which are used to optimize a multilayer perceptron (MLP) classifier. Instead of enforcing the classifier to strictly discriminate between positive and negative samples, we use a ranking-based loss (Tang *et al.* 2022) to guide the classifier to rank positive samples higher than unlabeled ones. Furthermore, since protein function is a multilabel classification problem, we rely on the GO hierarchical structure to construct class priors for each GO function (Fig. 1).

In this way, PU-GO aims to optimize a classifier in a more nuanced and accurate way for protein function prediction. This approach holds promise in enhancing the sensitivity and specificity of predictions, thereby contributing to a more comprehensive and reliable understanding of protein functions in complex biological systems. We show that PU-GO can outperform state-of-the-art protein function prediction methods in a similarity-based and time-based benchmark datasets.

## 2 Materials and methods

### 2.1 Positive–negative classification

Let $x\in R^{d}$ and y∈{−1,+1} be random variables with probability density function p(x,y) (du Plessis *et al.* 2014). Let $g:R^{d}\to R$ be an arbitrary decision function and $l:R\to R_{+}$ a loss function. The binary classifier *g* minimizes the risk:

$$R(g)=E_{\left(x,y)∼p(x,y\right)}[l(y\cdot g(x))]$$

where E is the expected value over p(x,y)

In standard binary classification, positive *P* and negative *N* datasets are given with distributions $p_{P}(x)=p(x|y=+1)$ and $p_{N}(x)=p(x|y=-1)$ (du Plessis *et al.* 2014). Given π=p(y=1) as the prior for *P*, the risk *R*(*g*) can be expressed as:

$$\begin{array}{c} R(g)=\pi R_{P}^{+}(g)+(1-\pi )R_{N}^{-}(g)\\ =\pi E_{x∼p_{P}(x)}[l(g(x))]+(1-\pi )E_{x∼p_{N}(x)}[l(-g(x))] \end{array}$$

Assuming data from *P* and *N* are sampled independently, *R*(*g*) can be approximated by:

(1) $$\hat{R}(g)=\pi {\hat{R}}_{P}^{+}(g)+(1-\pi ){\hat{R}}_{N}^{-}(g)$$

where ${\hat{R}}_{P}^{+}=\frac{-1}{\left|P\right|}\sum_{x\in P}l(g(x))$ and ${\hat{R}}_{N}^{-}=\frac{-1}{\left|N\right|}\sum_{x\in N}l(-g(x))$

### 2.2 PU classification

In PU classification, we assume the set *N* is empty and we are given an unlabeled dataset *U* with marginal probability density function p(x). In this case, the risk ${\hat{R}}_{N}$ cannot be computed. However, we can express ${\hat{R}}_{N}$ using the following equality (Plessis *et al.* 2015):

$$\begin{array}{c} \left(1-\pi )R_{N}^{-}(g)=R_{U}^{-}(g)-\pi R_{P}^{-}(g\right)\\ =E_{x∼p(x)}[l(-g(x))]-\pi E_{x∼p_{P}(x)}[l(-g(x))] \end{array}$$

and Equation (1) becomes:

(2) $$\hat{R}(g)=\pi {\hat{R}}_{P}^{+}(g)-\pi {\hat{R}}_{P}^{-}(g)+{\hat{R}}_{U}^{-}(g)$$

where ${\hat{R}}_{P}^{-}=\frac{-1}{\left|P\right|}\sum_{x\in P}l(-g(x))$ and ${\hat{R}}_{U}^{-}=\frac{-1}{\left|U\right|}\sum_{x\in U}l(-g(x))$. To avoid cases where *R*(*g*) can become negative, a non-negative estimator (Kiryo *et al.* 2017) is formulated as follows:

(3) $$\hat{R}(g)=\pi {\hat{R}}_{P}^{+}(g)+\max\{0,{\hat{R}}_{U}^{-}(g)-\pi {\hat{R}}_{P}^{-}(g)+\beta \}$$

where 0 ≤ β ≤ π. Since β ≤ π, we construct it using a margin factor hyperparameter *γ*, such that β=γπ, with 0 ≤ γ ≤ 1.

### 2.3 PU learning for function prediction

In the context of function prediction, the feature space for **x** and functions *l* and *g* must be defined. We use the ESM2 15B (Lin *et al.* 2023) model to generate vectors for protein sequences that are consequently used as feature space **x**. The ESM2 15B model generates vectors of size 5120 that we refer to as *ESM2 vectors*.

We implement the classifier *g* as a multilayer perceptron (MLP) that takes ESM2 vectors as inputs and returns values in $R^{k}$, where *k* is the number of classes. This classifier has shown to be effective in previous works (Kulmanov and Hoehndorf 2022). The MLP network contains two layers of MLP blocks where the output of the second MLP block has residual connection to the first block. This representation is passed to the final classification. One MLP block performs the following operations:

(4) MLPBlock(x)=DropOut(BatchNorm(ReLU(Wx + b)))

The input vector **x** of length 5120 represents ESM2 embedding and is reduced to 2048 by the first MLPBLock:

(5) h=MLPBlock(x)

This representation is passed to the second MLPBlock with the input and output size of 2048 and added to itself using residual connection:

(6) h=h + MLPBlock(h)

Finally, we pass this vector to a classification layer The output size of this layer is the same as the number of classes in each subontology:

(7) y=Wh + b

For PU learning, the loss function *l*(*x*) is:

(8) l(y)=ln(σ(y))

where $\sigma (x)=1/(1\;+\;e^{-x})$ is the sigmoid function.

### 2.4 Multilabel PU classification

Equation (3) computes a *binary* classification risk. Function prediction of proteins is a multilabel classification problem (i.e. each protein instance can be assigned multiple functions). Thus, given *k* GO functions, the classification risk must be minimized for all the GO functions. Therefore, the classifier *g* must minimize the following risk:

(9) $${\hat{R}}_{GO}(g)=\sum_{i=1}^{n}\pi_{i}{\hat{R}}_{P_{i}}^{+}(g)\;+\;\max\{0,{\hat{R}}_{U_{i}}^{-}(g)\;-\;\pi_{i}{\hat{R}}_{P_{i}}^{-}(g)\;+\;\beta \}$$

where *n* is the number of GO classes, *P_i_* (*U_i_*) is the set of positive (unlabeled) samples for the *i*th GO function.

Additionally, the factor $\pi_{i}=p(y_{i}=1)$ describe the prior probability of a protein being annotated with the *i*th GO function. Current approaches on estimating class priors have focused on leveraging instance similarity to identify potential positives in the unlabeled set (Zeiberg *et al.* 2020) or by subsampling positives and unlabeled instances to estimate the underlying distributions (Ramaswamy *et al.* 2016). In the context of function prediction, GO functions are structured hierarchically, which implies that all the proteins annotated to a function must also be annotated to the ontological ancestors of such function. We leverage this information to construct priors *π_i_* in the following way: we propagate annotations from each GO function to their ancestors and compute the frequency $S_{i}=N_{i}/N_{\mathrm{total}}$, where *N_i_* is the number of training proteins annotated with the *i*th GO function and *N*_total_ is the total number of training proteins. Let *S*_max_ be the largest frequency, then:

(10) $$\pi_{i}=\pi_{o}\cdot \frac{S_{i}}{S_{\max}}$$

where *π_o_* is a tunable hyperparameter. The rationale of computing priors based on frequency is that GO functions closer to the root of the hierarchy are more likely to be annotated due to the true-path rule that states that, if a protein *p* is annotated with class *C* and *C* is a descendant of *D* in the ontology, then *p* is also annotated with *D* (Ashburner *et al.* 2000).

### 2.5 Ranking positive and unlabeled samples

In Equation (9), ${\hat{R}}_{U_{i}}^{-}(g)=-\frac{1}{\left|U_{i}\right|}\sum_{x\in U_{i}}ln(\sigma (-g(x)))$. The term σ(−g(x)) pushed the scores to be 0, which may be unnecessarily difficult to achieve (Tang *et al.* 2022). An easier way to optimize the classifier *g* is to just push positive samples to be ranked higher than unlabeled samples. For this reason, we set:

(11) $${\hat{R}}_{U_{i}}^{-}(g)=-\frac{1}{\left|P_{i}|\cdot |U_{i}\right|}\sum_{x\in P_{i}}\sum_{y\in U_{i}}ln(\sigma (g(x)\;-\;g(y)))$$

Finally, the loss function in PU-GO is:

(12) $$L_{\text{PU-GO}}={\hat{R}}_{GO}(g)$$

### 2.6 UniProtKB/Swiss-Prot dataset and gene ontology

We use the dataset that was generated from manually curated and reviewed dataset of proteins from the UniProtKB/Swiss-Prot Knowledgebase (Consortium 2022) version 2023_03 released on 28 June 2023. We filtered all proteins with experimental functional annotations with evidence codes EXP, IDA, IPI, IMP, IGI, IEP, TAS, IC, HTP, HDA, HMP, HGI, HEP. The dataset contains 79, 973 reviewed and manually annotated proteins.

We split this dataset into training, validation and testing sets based on sequence similarity so that no similar sequences are shared between training, validation and testing sets. Our objective is to avoid over-fitting of our models to protein similarity. Therefore, we decided to split our dataset based on any similarity hit with maximum *e*-value score of 0.001. We computed pairwise similarity using Diamond (v2.0.9) (Buchfink *et al.* 2014), assigned sequences that have a similarity higher than our threshold to the same group, and split these groups into training (90%) and testing (10%). We extracted 10% of the training set to form a validation set. This resulted into a 81/9/10 split of the groups for training/validation/testing. We detail the split percentages in terms of proteins in Table 1. We call this dataset similarity-based dataset. We use Gene Ontology (GO) released on 01 January 2023. We train and evaluate models for each of the subontologies of GO separately.

**Table 1.** Summary of the UniProtKB/Swiss-Prot dataset.

Ontology	GO terms	Train (%)	Valid (%)	Test (%)	Time
MFO	7114	39 432 (89)	2359 (5)	2595 (6)	684
BPO	21 105	53 022 (89)	3180 (5)	3538 (6)	801
CCO	2888	51 991 (88)	3241 (6)	3565 (6)	573

The table shows the number of GO terms, number of proteins in similarity based training, validation and testing splits with percentages in parenthesis and the number of proteins in time-based evaluation benchmark dataset.

To compare our model with other methods we generated a test set by following the CAFA (Radivojac *et al.* 2013) challenge time-based approach. We downloaded UniProtKB/Swiss-Prot version 2024_01 released on 17 January 2024 and extracted newly annotated proteins in this version. Table 1 summarizes the datasets for each subontology.

### 2.7 Training procedure

To train our models, we optimized hyperparameters: batch size [30, 200], margin factor [0.1, 0.01], maximum learning rate [$10^{-2},5\cdot 10^{-6}$], minimum learning rate factor [$10^{-1},10^{-4}$], initial prior (*π_o_*) [$10^{-3},10^{-4}$]. Hyperparameters were optimized via Gaussian-Process Bayesian optimization method (Rasmussen and Williams 2005, Shahriari *et al.* 2016). We used Adam (Kingma and Ba 2015) optimizer and adapted the learning rate using a cyclic scheduler (Smith 2017). Selected hyperparameters can be found in the Supplementary Material.

### 2.8 Baseline and comparison methods

We trained PU-GO on the similarity-based dataset in order to avoid over-fitting to similar sequences. As baselines, we trained two baseline methods DeepGO-CNN (Kulmanov and Hoehndorf 2019) and DeepGOZero (Kulmanov and Hoehndorf 2022) and generate predictions without using any sequence similarity component such as BLAST (Altschul *et al.* 1997) or Diamond (Buchfink *et al.* 2014). For the time-based dataset evaluation we selected three state-of-the-art methods with openly available models as baseline: TALE (Cao and Shen 2021), SPROF (Yuan *et al.* 2023) and NetGO3 (Wang *et al.* 2023). Since baseline predictions also include sequence similarity components, we also combined PU-GO with Diamond by computing the arithmetic mean of the prediction scores of both methods:

(13) $$S_{\text{combined}}(p,f)=\frac{S_{\text{PU-GO}}(p,f)\;+\;S_{\mathrm{Diamond}}(p,f)}{2}$$

#### 2.8.1 Naive approach

Due to the imbalance in GO class annotations and propagation based on the true-path-rule, some classes have more annotations than others. Therefore, it is possible to obtain prediction results just by assigning the same GO classes to all proteins based on annotation frequencies. To test the performance obtained based on annotation frequencies, CAFA introduced a baseline approach called “naive” classifier (Radivojac *et al.* 2013). Here, each query protein *p* is annotated with the GO classes with a prediction scores computed as:

(14) $$S(p,f)=\frac{N_{f}}{N_{\mathrm{total}}}$$

where *f* is a GO class, *N_f_* is a number of training proteins annotated by GO class *f* and *N*_total_ is a total number of training proteins. We implement the same method.

#### 2.8.2 DiamondScore

The DiamondScore method is based on the sequence similarity score obtained by Diamond (Buchfink *et al.* 2014). The method aims to find similar sequences from the training set and transfer their annotations. We use the normalized bitscore to compute the prediction score for a query sequence *p*:

(15) $$S(p,f)=\frac{\sum_{s\in E}\text{bitscore}(p,s)\cdot I(f\in T_{s})}{\sum_{s\in E}\text{bitscore}(p,s)}$$

where *E* is a set of similar sequences filtered by *e*-value of 0.001, *T_s_* is a set of true annotations of a protein with sequence *s*, and *I* is an indicator function that returns 1 if the condition is true and 0 otherwise.

#### 2.8.3 MLP (ESM2)

The MLP baseline method predicts protein functions using a multilayer perceptron (MLP) from a protein’s ESM2 embedding (Lin *et al.* 2023). We generate an embedding vector of size 5192 using ESM2 15B model and pass it to the MLP described in Equation (4). Additionally, we pass this representation to a sigmoid activation function.

(16) y=σ(y)

We train a different model for each subontology in GO.

#### 2.8.4 DeepGO-plus and DeepGOCNN

DeepGO-PLUS (Kulmanov and Hoehndorf 2019) predicts function annotations of proteins by combining DeepGOCNN, which predicts functions from the amino acid sequence of a protein using a 1-dimensional convolutional neural network (CNN), with the DiamondScore method. DeepGOCNN captures sequence motifs that are related to GO functions. Here, we only use CNN based predictions.

#### 2.8.5 DeepGOZero

DeepGOZero (Kulmanov and Hoehndorf 2022) combines protein function prediction with a model-theoretic approach for embedding ontologies into a distributed geometric space. ELEmbeddings (Kulmanov *et al.* 2019) represent classes as *n*-balls and relations as vectors to embed ontology semantics into a geometric model. It uses InterPro domain annotations represented as binary vector as input and applies two layers of MLPBlock as in our MLP baseline method to generate an embedding of size 1024 for a protein. It learns the embedding space for GO classes using ELEmbeddings loss functions and optimizes together with protein function prediction loss. For a given protein *p* DeepGOZero predicts annotations for a class *c* using the following formula:

(17) $$y_{c}^{\prime }=\sigma (f_{\eta }(p)\cdot {\left(f_{\eta }(hF)\;+\;f_{\eta }(c)\right)}^{T}\;+\;r_{\eta }(c))$$

where $f_{\eta }$ is an embedding function, *hF* is the hasFunction relation, $r_{\eta }(c)$ is the radius of an *n*-ball for a class *c* and *σ* is a sigmoid activation function. It optimizes binary crossentropy loss between predictions and the labels together with ontology axioms losses from ELEmbeddings.

#### 2.8.6 TALE

TALE (Cao and Shen 2021) predicts functions using a transformer-based deep neural network model which incorporates hierarchical relations from the GO into the model’s loss function. The deep neural network predictions are combined with predictions based on sequence similarity. We used the trained models provided by the authors to evaluate them on the time-based dataset.

#### 2.8.7 SPROF-GO

SPROF-GO (Yuan *et al.* 2023) method uses the ProtT5-XL-U50 (Elnaggar *et al.* 2022) protein language model to extract proteins sequence embeddings and learns an attention-based neural network model. The model incorporates the hierarchical structure of GO into the neural network and predicts functions that are consistent with hierarchical relations of GO classes. Furthermore, SPROF-GO combines sequence similarity-based predictions using a homology-based label diffusion algorithm. We used the trained models provided by the authors to evaluate them on the time-based dataset.

#### 2.8.8 NetGO3

NetGO3 integrates seven component methods that differ on the type of information they rely on: (1) Naive: GO frequency, (2) BLAST-KNN: sequence homology, (3) LR-3mer: amino acid trigram, (4) LR-InterPro: domain/family/motif, (5) NetKNN: protein network, (6) LR-Text: literature, and (7) LR-ESM: protein language model. Methods with the prefix “LR” and “KNN”contain a logistic regression classifier and k-nearest neighbor algorithm, respectively. We used the web service provided by the authors to obtain predictions for our time-based benchmark dataset.

#### 2.8.9 ATGO

ATGO (Zhu *et al.* 2022) uses the ESM-1b protein language model. For a protein sequence, the model extracts embeddings from the three last layers of ESM-1b. The embeddings are inputs for an MLP-based neural network. ATGO computes a triplet loss, which means that for an anchor protein *anc*, proteins *pos* and *neg* are sampled with the same or different functions as *anc*, respectively. The final model, ATGO+, combines the prediction scores of ATGO with a sequence homology based method. We used the trained models provided by the authors to evaluate ATGO+ on the time-based dataset.

### 2.9 Evaluation

We use four different measures to evaluate the performance of our models. Three protein-centric measures $F_{\max},S_{\min}$, and AUPR and one class-centric AUC.

$F_{\max}$ is a maximum protein-centric *F*-measure computed over all prediction thresholds. First, we compute average precision and recall using the following formulas:

(18) $$pr_{i}(t)=\frac{\sum_{f}I(f\in P_{i}(t)\wedge f\in T_{i})}{\sum_{f}I(f\in P_{i}(t))}$$

(19) $$rc_{i}(t)=\frac{\sum_{f}I(f\in P_{i}(t)\wedge f\in T_{i})}{\sum_{f}I(f\in T_{i})}$$

(20) $$\mathrm{AvgPr}(t)=\frac{1}{m(t)}\cdot \sum_{i=1}^{m(t)}pr_{i}(t)$$

(21) $$\mathrm{AvgRc}(t)=\frac{1}{n}\cdot \sum_{i=1}^{n}rc_{i}(t)$$

where *f* is a GO class, *T_i_* is a set of true annotations, $P_{i}(t)$ is a set of predicted annotations for a protein *i* and threshold *t*, *m*(*t*) is a number of proteins for which we predict at least one class, *n* is a total number of proteins and *I* is an indicator function which returns 1 if the condition is true and 0 otherwise. Then, we compute the $F_{\max}$ for prediction thresholds t∈[0,1] with a step size of 0.01. We count a class as a prediction if its prediction score is greater or equal than *t*:

(22) $$F_{\max}=\max_{t}\left\{\frac{2\cdot \mathrm{AvgPr}(t)\cdot \mathrm{AvgRc}(t)}{\mathrm{AvgPr}(t)\;+\;\mathrm{AvgRc}(t)}\right\}$$

$S_{\min}$ computes the semantic distance between real and predicted annotations based on information content of the classes. The information content *IC*(*c*) is computed based on the annotation probability of the class *c*:

(23) IC(c)=−log(Pr(c|P(c))

where *P*(*c*) is a set of parent classes of the class *c*. The $S_{\min}$ is computed using the following formulas:

(24) $$S_{\min}=\min_{t}\sqrt{ru{\left(t\right)}^{2}\;+\;mi{\left(t\right)}^{2}}$$

where *ru*(*t*) is the average remaining uncertainty and *mi*(*t*) is average misinformation:

(25) $$ru(t)=\frac{1}{n}\sum_{i\;=\;1}^{n}\sum_{c\in T_{i}\;-\;P_{i}(t)}IC(c)$$

(26) $$mi(t)=\frac{1}{n}\sum_{i\;=\;1}^{n}\sum_{c\in P_{i}(t)\;-\;T_{i}}IC(c)$$

AUPR is the area under the average precision (*AvgPr*) and recall (*AvgRc*) curve. AUC is a class-centric measure where compute AUC ROC per each class and take the average.

## 3 Results

### 3.1 Prediction model: PU-GO

We developed PU-GO, a method based on positive unlabeled learning to predict GO functions. PU-GO acts on the MLP classifier shown in Equations (5–8). The training phase uses the output of the classifier to compute the classification risk of positive and unlabeled samples following Equation (9). In the prediction phase, the output of the classifier is passed to the sigmoid function directly.

We trained three separate models for each subontology. The only parametric difference between the three models is the output size of the classifier, which depends of the number of GO functions. For Molecular Function Ontology there are 7114 functions, for Cellular Component ontology 2888 and for Biological Process Ontology 21 105.

We used the similarity-based dataset to train our models in order to avoid bias induced by sequence-similar proteins existing in training and testing datasets. For each model, we trained 10 models varying the random seed used to initialized model parameters and batch selection and aggregated the metrics using the arithmetic mean operation.

### 3.2 Evaluation on similarity-based split

To evaluate PU-GO, we chose baseline methods that do not contain components relying on sequence similarity for computing prediction scores. Results are shown in Table 2. PU-GO outperforms other methods in almost all evaluations except in AUPR in BPO, where MLP(ESM2) obtains the best performance. However, it is possible that using ESM2 15B in PU-GO is the reason to outperform DeepGO-CNN and DeepGOZero. The advantage of PU learning is directly demonstrated when comparing PU-GO to MLP(ESM2) which uses the same classifier function as PU-GO but considers unlabeled samples as negatives.

**Table 2.** Evaluation results for similarity-based split using protein-centric $F_{\max},\;S_{\min}$, and AUPR, and the class-centric average AUC.

Method	$F_{\max}$	$S_{\min}$	AUPR	AUC
MFO				
Naive	0.2805	15.1460	0.1395	0.5000
DeepGO-CNN	0.3705	14.1480	0.3242	0.7087
DeepGOZero	0.4545	12.8750	0.4095	0.7536
MLP(ESM2)	0.5079	12.1020	0.4851	0.8401
PU-GO	**0.5317**	**11.6490**	**0.5026**	**0.8413**
BPO				
Naive	0.2997	41.6290	0.1978	0.5000
DeepGO-CNN	0.3446	40.4210	0.2810	0.6879
DeepGOZero	0.3624	39.5340	0.3112	0.6900
MLP(ESM2)	0.4279	37.1990	**0.3973**	0.8484
PU-GO	**0.4365**	**36.7640**	0.3928	**0.8674**
CCO				
Naive	0.5501	12.3280	0.4077	0.5000
DeepGO-CNN	0.6336	11.2260	0.6343	0.7699
DeepGOZero	0.5862	11.8080	0.5711	0.6836
MLP(ESM2)	0.7091	9.4250	0.6897	0.9047
PU-GO	**0.7210**	**9.1010**	**0.7696**	**0.9240**

Bold values indicate best performance.

### 3.3 Evaluation on time-based benchmark

To test the generalization capability of PU-GO, we use our trained models optimized using data from UniProtKB/SwissProt Knowledgebase version 2023_03, to predict GO functions from UniProtKB/SwissProt Knowledgebase version 2024_01. We compared with several state-of-the-art methods and show the results in Table 3. We integrate Diamond predictions with PU-GO as shown in Equation (13). PU-GO+Diamond outperforms all methods in the class-centric AUC evaluation across all subontologies and obtains the highest $F_{\max}$ in BPO and CCO. However, ATGO+ resulted in best $F_{\max}$ and $S_{\min}$ in MFO.

**Table 3.** Evaluation results for time-based split using protein-centric $F_{\max},\;S_{\min}$, and the class-centric average AUC.

Method	$F_{\max}$	$S_{\min}$	AUC
MFO			
Diamond	0.555	8.915	0.848
SPROF-GO	0.539	9.482	0.724
TALE	0.301	13.515	0.741
NetGO3	0.539	8.986	0.891
ATGO+	**0.612**	**8.061**	0.844
MLP(ESM2)	0.507	9.682	0.942
PU-GO	0.531	9.010	0.947
MLP(ESM2)+Diamond	0.562	8.948	0.953
PU-GO+Diamond	0.569	8.776	**0.955**
BPO			
Diamond	0.548	23.693	0.735
SPROF-GO	0.496	24.368	0.722
TALE	0.476	25.612	0.644
NetGO3	0.542	23.061	0.697
ATGO+	0.549	**22.156**	0.736
MLP(ESM2)	0.448	26.481	0.884
PU-GO	0.471	25.175	0.888
MLP(ESM2)+Diamond	0.550	23.532	0.902
PU-GO+Diamond	**0.556**	22.993	**0.904**
CCO			
Diamond	0.685	7.786	0.792
SPROF-GO	0.711	7.729	0.777
TALE	0.664	8.685	0.785
NetGO3	0.680	7.964	0.897
ATGO+	0.712	7.535	0.822
MLP(ESM2)	0.681	8.413	0.931
PU-GO	0.701	7.866	0.945
MLP(ESM2)+Diamond	0.725	7.340	0.937
PU-GO+Diamond	**0.734**	**7.062**	**0.949**

Bold values indicate best performance.

**Figure 2.**
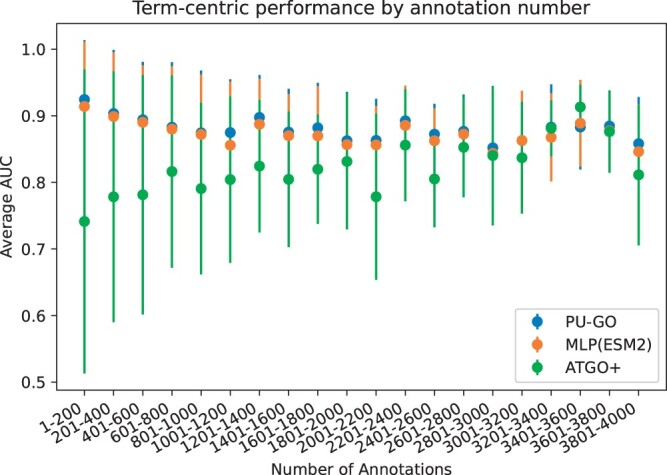
Average prediction performance of GO classes grouped by number of annotations on UniprotKB-Swissprot dataset.

We further analyze the performance of MLP(ESM2), PU-GO, and ATGO+ based on the specificity of the GO class. Less specific GO classes are closer to the root and contain a higher number of protein annotations than classes with high specificity. We compute the AUC for each GO class and group them by their number of annotations. We find that positive unlabeled learning in PU-GO improves the performance across all levels of specificity when compared with MLP(ESM2). Regarding ATGO+, we find that its average AUC is lower than PU-GO+Diamond for GO classes with higher level of specificity, whereas it can outperform PU-GO+Diamond for GO classes with lower level of specificity (i.e. larger number of annotations) (Fig. 2).

### 3.4 Ablation study

PU-GO contains two variations from the standard PU learning formulation such as (1) the use of a ranking loss between positive and unlabeled samples following (Tang *et al.* 2022) and (2) the use of a different prior for each GO class using GO hierarchical structure. We analyze the impact of each component in Table 4. PU-basic uses Equation (9) with $\pi_{i}=\pi_{o}$ for every *i*th GO function. From PU-basic, we construct PU-ranking replacing the risk estimation for unlabeled samples ${\hat{R}}_{U_{i}}^{-}$ from Equation (9) with a risk computing the ranking between positive and unlabeled samples in Equation (11). PU-ranking is more flexible than PU-basic, and only requires unlabeled samples to be scored lower than positive ones and not strictly close to 0, which results in better performance in general. Finally, from PU-ranking we construct PU-GO by incorporating custom priors *π_i_* for each GO class [Equation (10)]. This change shallowly incorporates hierarchy information as class priors (i.e, a GO class closer to the root is more likely to be annotated with a protein than a GO class closer to the leaves). Our analysis shows that using custom prior values enhance PU learning. For every method, we trained 10 models with different random seeds and report the arithmetic mean and standard deviation values.

**Table 4.** Ablation study analyzing the components of PU-GO.

Method	$F_{\max}$	$S_{\min}$	AUPR	AUC
MFO				
PU-basic	0.5129 ± 0.0041	11.9470 ± 0.0696	0.4319 ± 0.0046	0.8446 ± 0.0054
PU-ranking	0.5265 ± 0.0014	11.7450 ± 0.0414	0.4556 ± 0.0067	**0.8510 ± 0.0028**
PU-GO	**0.5317 ± 0.0020**	**11.6490 ± 0.0471**	**0.5026 ± 0.0036**	0.8413 ± 0.0062
BPO				
PU-basic	0.4310 ± 0.0005	37.0440 ± 0.0482	0.3655 ± 0.0018	0.8602 ± 0.0020
PU-ranking	**0.4368 ± 0.0007**	**36.7090 ± 0.0602**	**0.4086 ± 0.0033**	**0.8677 ± 0.0011**
PU-GO	0.4365 ± 0.0008	36.764 ± 0.0364	0.3928 ± 0.0009	0.8674 ± 0.0023
CCO				
PU-basic	0.6994 ± 0.0007	9.6110 ± 0.0384	0.6152 ± 0.0012	0.9039 ± 0.0053
PU-ranking	0.7102 ± 0.0007	9.4060 ± 0.0200	0.6239 ± 0.0014	0.8933 ± 0.0072
PU-GO	**0.7210 ± 0.0010**	**9.1010 ± 0.0244**	**0.7696 ± 0.0043**	**0.9240 ± 0.0018**

Metrics reported are protein-centric $F_{\max}$, $S_{\min}$, and AUPR, and the class-centric average AUC.

Bold values indicate best performance.

## 4 Discussion

Positive-unlabeled learning is an appropriate formulation to the automated function prediction problem, where most of the data is still not labeled. Previous attempts to handle unlabeled data aim to transform some unlabeled samples into negatives (Youngs *et al.* 2013) or have not been applied to current deep learning classifiers (Song *et al.* 2021). We developed PU-GO, adapting *risk-minimization* based PU learning (Elkan and Noto 2008, du Plessis *et al.* 2014, Plessis *et al.* 2015, Kiryo *et al.* 2017, Bekker and Davis 2020) to the context of function prediction. PU-GO does not require extracting a subset of unlabeled samples as negatives. Instead, the whole unlabeled dataset can be used to adapt a classifier.

PU learning with risk-minimization framework is a function of a classifier. In our case, we used an MLP classifier. The input for the MLP were vectors from ESM2 15B, a pretrained language model for protein sequences. This configuration (i.e. ESM2 15B + MLP) is similar to other methods such as SPROF-GO (Yuan *et al.* 2023), NetGO3 (Wang *et al.* 2023), which as part of their frameworks there are pretrained language models together with a classifier. PU-GO does not contain any additional component other than the ESM2 15B+MLP classifier. We showed that PU-GO was able to outperform baseline methods as well as the binary classification training version of ESM2 15B + MLP, which supports the hypothesis that PU learning is an appropriate approach to improve protein function prediction. However, more sophisticated classifiers can be proposed in future work, where incorporation of additional domain-specific biological data can be used to constrain the optimization process.

Class prior estimation is a crucial aspect in PU learning (du Plessis *et al.* 2016). For protein function prediction, we leveraged domain-specific information such as the GO hierarchical structure to design custom class priors per each GO class based on their annotation frequency. Our approach requires tuning an initial prior weight *π_o_*, which we selected empirically by searching in a particular range. Despite the simplicity of this approach, it showed to be effective to construct a more robust models. However, future work can explore other ways to construct more accurate priors by leveraging other aspects of GO such as semantic similarity between GO classes instead of only using class annotation frequency as in PU-GO. Similarly, other class prior estimation strategies should be explored, such as instance-similarity-based (Zeiberg *et al.* 2020) or positive-unlabeled subsampling (Ramaswamy *et al.* 2016). Furthermore, biological information can also be leveraged to construct better class priors such as protein sequence homology (Yuan *et al.* 2023).

PU-GO framework handles unlabeled samples differently than previous approaches where the aim was to strictly discriminate between positive and negative samples. In PU-GO, instead of minimizing the risk of classifying an unlabeled sample as negative, it addresses the protein function prediction as a ranking problem and minimizes the risk of ranking an unlabeled sample higher than a positive one. Furthermore, since the risk-minimization framework we resort to is extensible to incorporate true negative samples (Hsieh *et al.* 2019), future work can be directed to study the incorporation of negative annotations that are already available or that can be extracted by some strategy.

## 5 Conclusion

Protein function prediction is a widely studied multilabel classification problem that typically has been addressed under binary classification settings. However, protein function annotations are mostly unlabeled. To deal with unlabeled annotations, we addressed protein function prediction as a PU classification problem. We adapted the PU learning framework for protein function prediction by incorporating hierarchical information in GO in the class priors. Our analysis indicates improved performance compared to existing methods on similarity-based and time-based benchmark datasets. Future potential work could focus on incorporating negative samples to the PU setting and minimize negative classification risk. Although negative data is small, finding a way to use it can improve the classifier generalization capability. Another direction could be using more sophisticated classifiers that can include other types of biological information, which has been an approach followed in the binary-classification setting.

## Supplementary Material

btae237_Supplementary_Data

## Data Availability

The data underlying this article are available in a Zenodo Repository at https://dx.doi.org/10.5281/zenodo.11079885.
